# Differential interleukin-1β induction by uropathogenic *Escherichia coli* correlates with its phylotype and serum C-reactive protein levels in Korean infants

**DOI:** 10.1038/s41598-019-52070-3

**Published:** 2019-10-30

**Authors:** Jong-Hyeok Jung, Hyun Jung Hong, Aziz Gharderpour, Jae Young Cho, Bum-Seo Baek, Yong Hur, Byoung Choul Kim, Donghyun Kim, Seung-Yong Seong, Jae-Young Lim, Sang-Uk Seo

**Affiliations:** 1 Department of Biomedical Sciences, Seoul National University College of Medicine, Seoul, Republic of Korea; 2 Wide River Institute of Immunology, Seoul National University College of Medicine, Hongcheon, Gangwon-do, Republic of Korea; 3 Department of Pediatrics, Gyeongsang National University School of Medicine, Jinju, Gyeongsangnam-do Republic of Korea; 4 Gyeongsang Institute of Health Science, Jinju, Gyeongsangnam-do Republic of Korea; 5 Department of Medicine, Seoul National University College of Medicine, Seoul, Republic of Korea; 6 Division of Nano-bioengineering, Incheon National University, Incheon, Republic of Korea; 7 Department of Microbiology and Immunology, Institute of Endemic Diseases, Seoul National University College of Medicine, Seoul, Republic of Korea; 8 Mucosal Immunology Laboratory, University of Ulsan College of Medicine, Seoul, Republic of Korea

**Keywords:** Infectious-disease epidemiology, Bacteriology, Urinary tract infection

## Abstract

Urinary tract infection (UTI) is one of the most common bacterial infections in infants less than age 1 year. UTIs frequently recur and result in long-term effects include sepsis and renal scarring. Uropathogenic *Escherichia coli* (UPEC), the most prevalent organism found in UTIs, can cause host inflammation via various virulence factors including hemolysin and cytotoxic necrotizing factors by inducing inflammatory cytokines such as interleukin (IL)-1β. However, the ability of each UPEC organism to induce IL-1β production may differ by strain. Furthermore, the correlation between differential IL-1β induction and its relevance in pathology has not been well studied. In this study, we isolated UPEC from children under age 24 months and infected bone-marrow derived macrophages with the isolates to investigate secretion of IL-1β. We found that children with higher concentrations of C-reactive protein (CRP) were more likely to harbor phylotype B2 UPEC strains that induced more IL-1β production than phylotype D. We also observed a significant correlation between serum CRP level and *in vitro* IL-1β induction by phylotype B2 UPEC bacteria. Our results highlight the diversity of UPEC in terms of IL-1β induction capacity in macrophages and suggest a potential pathogenic role in UTIs by inducing inflammation in infants.

## Introduction

Urinary tract infection (UTI) is one of the most common bacterial infections and occurs in approximately 150 million people a year^1^. Infants under age 1 year are more susceptible to UTIs. In general, UTIs are more common in girls, although before age 1 year boys have more UTIs than girls^2^. A previous study found that 18% of infants under age 12 months who experience UTIs have recurrences within a few months^3^. Moreover, depending on when a UTI is diagnosed and treated after occurrence, the outcome may include sepsis, renal scarring, and hypertension^4^.

Among bacteria that can lead to UTI, uropathogenic *Escherichia coli* (UPEC) is the most common and is found in 80~90% of UTI patients^1^. As the urinary tract is a harsh environment for bacteria due to continuous flow of urine, UPECs can replicate in the form of intracellular bacterial communities (IBCs) as a strategy to survive^5^. Various virulence factors such as fimbriae/adhesins, pore-forming toxins, and iron-uptake molecules contribute to this survival strategy^6^. UPEC make use of fimbriae and adhesins, including type 1 fimbriae, P fimbriae, and Afa adhesins, to adhere to host cell surfaces^7,8^ while pore-forming toxins such as cytotoxic necrotizing factors (CNF) and hemolysin of UPEC can make pores in host cell membranes for invasion^9,10^.

Inflammation often accompanies UTIs and is associated with renal scarring and disease severity^11^. Several cytokines, including TNF-α, interleukin (IL)−1β, IL-6, and IL-8, are involved in the inflammation that accompanies UTIs^12^. IL-1β, which is often detected in serum samples of children with UTIs, has been used as a marker for acute pyelonephritis^13^. IL-1β is primarily secreted by monocytes and macrophages. It induces tissue damage and infiltration of neutrophils. To avoid uncontrolled inflammation, secretion of the active-form of IL-1β is tightly regulated and modulated by a molecular complex called inflammasome^14^. Several UPEC virulence factors can activate inflammasome and directly influence IL-1β secretion. Pore-forming toxin, especially hemolysin, is known to induce IL-1β secretion and cell death in bladder tissue^15^. Hemolysin of *Proteus mirabilis* and group B *Streptococcus* can also induce IL-1β secretion via NLRP3 inflammasome and enhance inflammation^16,17^. In UTI and meningitis animal models, *E. coli* CNF exacerbates inflammation^18,19^. CNF can synergistically promote IL-1β secretion with lipopolysaccharide in a caspase-1/caspase-11-dependent manner^20^.

Macrophages in the urinary tract have various roles in host defense against invading UPEC. At an early infection time point, the absence of macrophage results in a higher bacterial burden and alteration of innate immune signaling^21^. Macrophages in urinary tissue can recruit neutrophils to the uroepithelium during UPEC infection and depletion of these tissue macrophages results in ablation of neutrophil migration and bacteria clearance^22^. Moreover, there is evidence that some UPEC strains can directly infect macrophages, reside in intracellular vesicles, and make IBCs^23,24^. Invasion of macrophages by UPEC can result in prolonged survival of UPEC and recurrent infection^25^. From these results, we can consider macrophages in the urinary tract to be both sensor and reservoir of UPEC.

Much research about inflammation in UTI has focused on host responses at the tissue level or interactions between UPEC and epithelial cells^26–28^. However, myeloid cells recruited to the site of infection also play an immediate role in innate immune responses^21,29^. Macrophages are not only target cells for primary UPEC infection but they also play key roles in inflammatory response^23^. Inflammation is considered a double-edged sword in many diseases because it is essential for controlling infection while it is hazardous to the host when exacerbated in the acute phase^30–32^. To investigate the relationship between different characteristics of UPEC and a patient’s inflammatory responses, we recruited patients under age 24 months and measured serum C-reactive protein (CRP) concentration, which is known to rise in response to inflammation. We chose this age group because infants are highly susceptible to severe progression including renal scarring upon UTI^33^. In parallel, we isolated UPEC strains from child patients to analyzed phylotype, virulence gene expression, and IL-1β induction potential. Integrative analyses were made from a data series obtained from patient’s blood and UPEC isolates.

## Results

### Subject characteristics

We analyzed 40 *E. coli* isolates from individual children. The median age of the participants was 4.7 months (range, 0.4–16.7) and 27% (11/40) were girls. The mean white blood cell (WBC) count of these patients was 15,279/mm³. All had fever ≥38 °C, pyuria, and *E. coli* identified in urine cultures. Mean CRP levels were 44.5 mg/L (range, 0.9–189.5). Twenty-two children had CRP levels ≥30 mg/L. A DMSA (dimercaptosuccinic acid) scan showed cortical defects in 8 of the 40 patients. Children with CRP levels ≥30 mg/L were more likely to have defects shown by DMSA (36.3% vs. 0%, *p* = 0.005). Vesicoureteral reflux (VUR) was found in 5 of 23 patients who underwent a voiding cystourethrogram (VCUG). Ultrasonography (USG) revealed 5 patients with hydronephrosis, 9 with cystitis, and 2 with acute pyelonephritis (Table 1).

**Table 1:** Characteristics of study population with urinary tract infections.

	CRP < 30 (n = 18)	CRP ≥ 30 (n = 22)	All (n = 40)	*p*-value
Median age, mo (Max-Min)	3.7 (0.5–7.9)	5.5 (0.4–18.0)	4.7 (0.4–18.0)	0.004
Girls, n (%)	6 (33.3)	5 (22.7)	11 (27.5)	0.498
WBC, /mm³ (Max-Min)	13927 (4470–24580)	16438 (7610–25090)	15279 (4470–25090)	0.235
CRP, mg/L (Max-Min)	12.5 (0.9–29.4)	70.7 (32.8–189.5)	44.5 (0.9–189.5)	0.000
Cortical defect in DMSA, n (%)	0/18 (0.0)	8/22 (36.3)	8/40 (20.0)	0.005
VUR, n (%)	2/13 (15.5%)	3/10 (30.0)	5/23 (21.7)	0.39
**Abnormal findings on sonography**				
Hydro, n	2/18	3/20	5/38	1.00
APN, n	2/18	0/20	2/38	0.21
Cystitis, n	4/18	5/20	9/38	1.00

Note: APN, acute pyelonephritis; CRP, C-reactive protein; DMSA, dimercaptosuccinic acid; Hydro, hydronephrosis D; Max, maximum; Min, minimum; VUR, vesicoureteral reflux; WBC, white blood cells.

### Phylotype and pathotypic distribution of UPEC isolates

All isolates were identified as *E. coli* by *phoA* gene-specific amplification (Supplementary Table 1). Confirmed strains were used to infect bone marrow-derived macrophage (BMDM) for further studies, including phylotypic and pathotypic analysis. When phylotyping was performed^34^, among 40 isolates, 28 (70%) were group B2 *E. coli* while 12 (30%) were group D. No group A or B1 strains were isolated (Table 2).

**Table 2:** Phylotypic and virulence gene distribution of UTI bacteria in 40 children.

Distribution of virulence genes	Phylogenetic group		Total (n = 40), %
	Group B2 (n = 28), %	Group D (n = 12), %	
*papC*	18 (64.3)	7 (58.3)	25 (62.5)
*sfa/foc (sfa)*	6 (21.4)	—	6 (15)
*afaC*	6 (21.4)	1 (8.3)	7 (17.5)
*fimH*	20 (71.4)	11 (91.7)	31 (77.5)
*cnf*	3 (10.7)	—	3 (7.5)
*hly*	4 (14.3)	—	4 (10)
*aer*	15 (53.6)	10 (83.3)	25 (62.5)

Note: No Group A or B1 genes were found.

To determine pathotypic characteristics of UPEC isolates, we investigated the presence of seven virulence genes by polymerase chain reaction (PCR). Of these, *papC* were found in 25 (62.5%), *sfa/focDE* in 6 (15%), *afaC* in 7 (17.5%), *fimH* in 31 (44.5%), *cnf* in 3 (7.5%), *hlyCA* in 4 (10%), and *iucC* in 25 (62.5%). All virulence genes were more prevalent in group B2 *E. coli*. Six of 7 *afaC-*positive strains were group B2 while *sfa/focDE, cnf, hlyCA* genes were only detected from group B2 (Table 2).

### Cytokine secretion profile and viability of BMDM upon infection of UPEC strains

**Figure 1:**
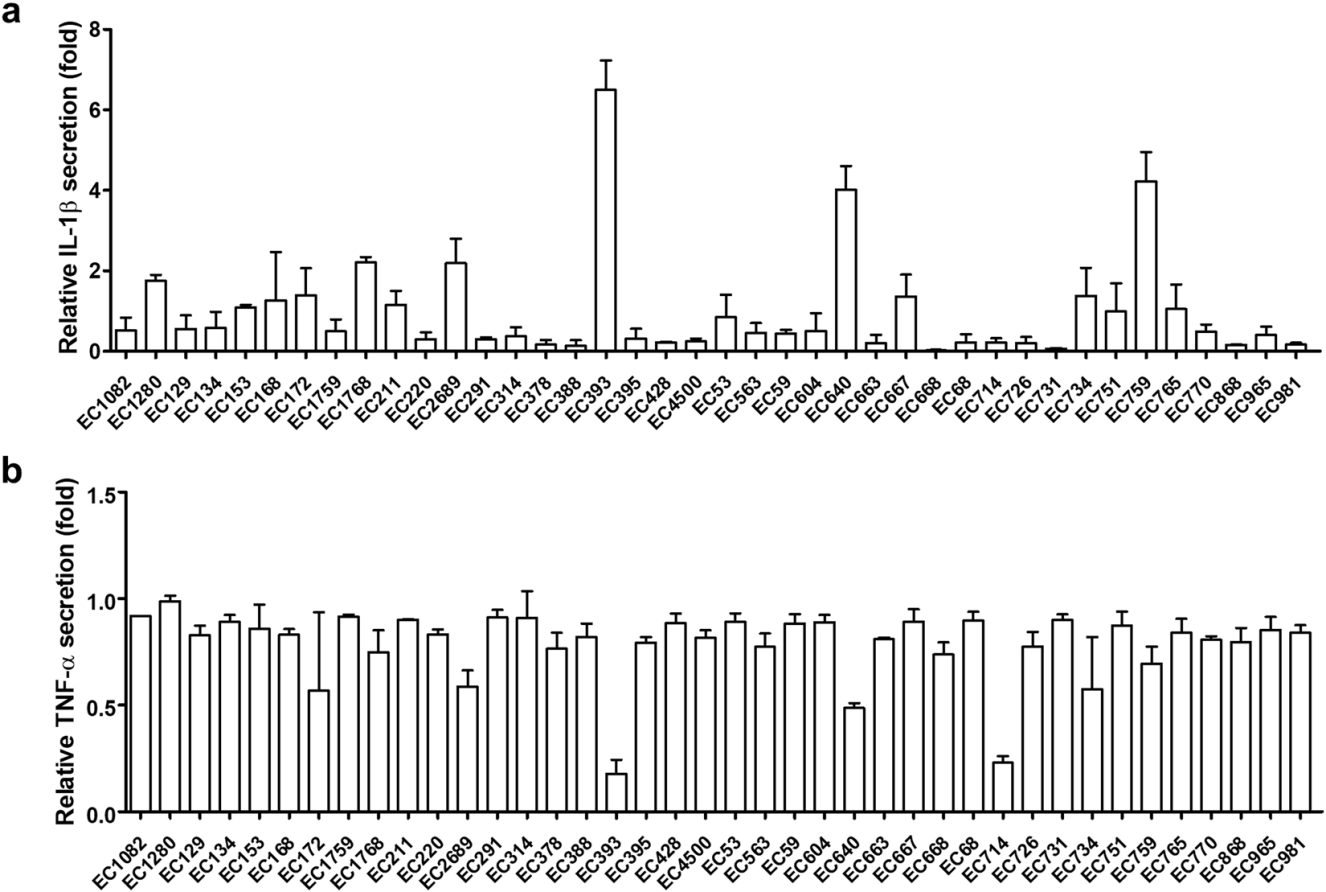
UPEC isolates induce IL-1β from BMDM at different levels. BMDMs were co-cultured with UPEC isolates for 12 h. Levels of (**a**) IL-1β and (**b**) TNF-α in culture supernatants were measured. Amounts of cytokines induced by UPEC isolates were normalized against cytokines produced by *P. mirabilis* stimulation. Values represent mean ± SD from six samples from two separate experiments.

**Figure 2:**
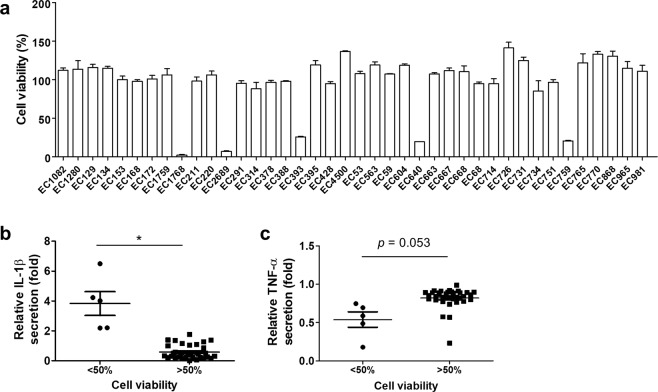
Cytotoxic UPEC induces higher IL-1β and lower TNF-α levels than other UPEC strains. (**a**) BMDMs were co-cultured with UPEC isolates for 12 h and cell viability was measured. Isolates were divided into lower and higher viability groups (<50% and >50%, respectively) and their induced secretion of (**b**) IL-1β and (**c**) TNF-α from BMDM after co-culture was compared. Cytokine levels were normalized against those of a *P. mirabilis* stimulated group. Each dot represents a mean of six samples from two independent experiments. *p < 0.05.

To examine cytokine secretion by BMDM co-cultured with UPEC isolates, we performed ELISA on culture medium 12 h after stimulation. Because *P. mirabilis* can efficiently induce IL-1β and TNF-α production in BMDM^17^, we used this strain as a positive control. Among 40 isolates, 5 exhibited more than two-fold higher IL-1β secretion than *P. mirabilis* (Fig. 1a). In many instances, TNF-α levels did not differ significantly among isolates. However, three strains were reduced by more than half compared with *P. mirabilis* (Fig. 1b). IL-1β can induce pyroptosis, a type of cell death that results in membrane rupture and release of inflammatory components^35^. Therefore, we investigated cell viability of infected BMDMs to examine cell death (Fig. 2a). Co-culture of BMDM with five isolates resulted in reduced cell viability. When we assessed the correlation between cytokine production and cell viability, the top five IL-1β-secreting isolates had obvious reduction (*p* = 0.015) in cell viability (Fig. 2b). These five strains also showed reduced production of TNF-α (Fig. 1a,b). However, the correlation between TNF-α and cell viability did not reach statistical significance (Fig. 2c). These data suggest that UPEC strains with more cytotoxicity can induce more IL-1β secretion *in vitro*.

### Group B2 UPEC is associated with IL-1β induction *in vitro* and patient’s serum CRP level

**Figure 3:**
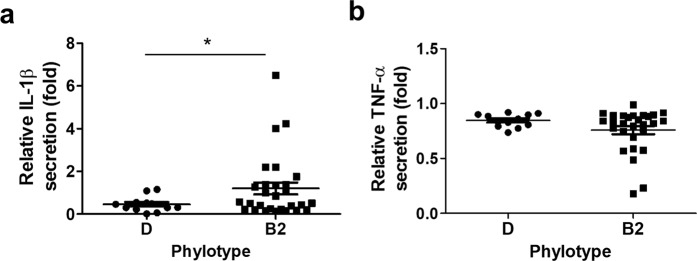
Group B2 UPECs are more likely to induce IL-1β from BMDM than are group D UPECs. Phylotypes of UPEC isolates were determined. (**a**) IL-1β and (**b**) TNF-α induction data were re-grouped and analyzed based on phylotypes. Each dot represents a mean of six samples from two independent experiments. *p < 0.05.

**Figure 4:**
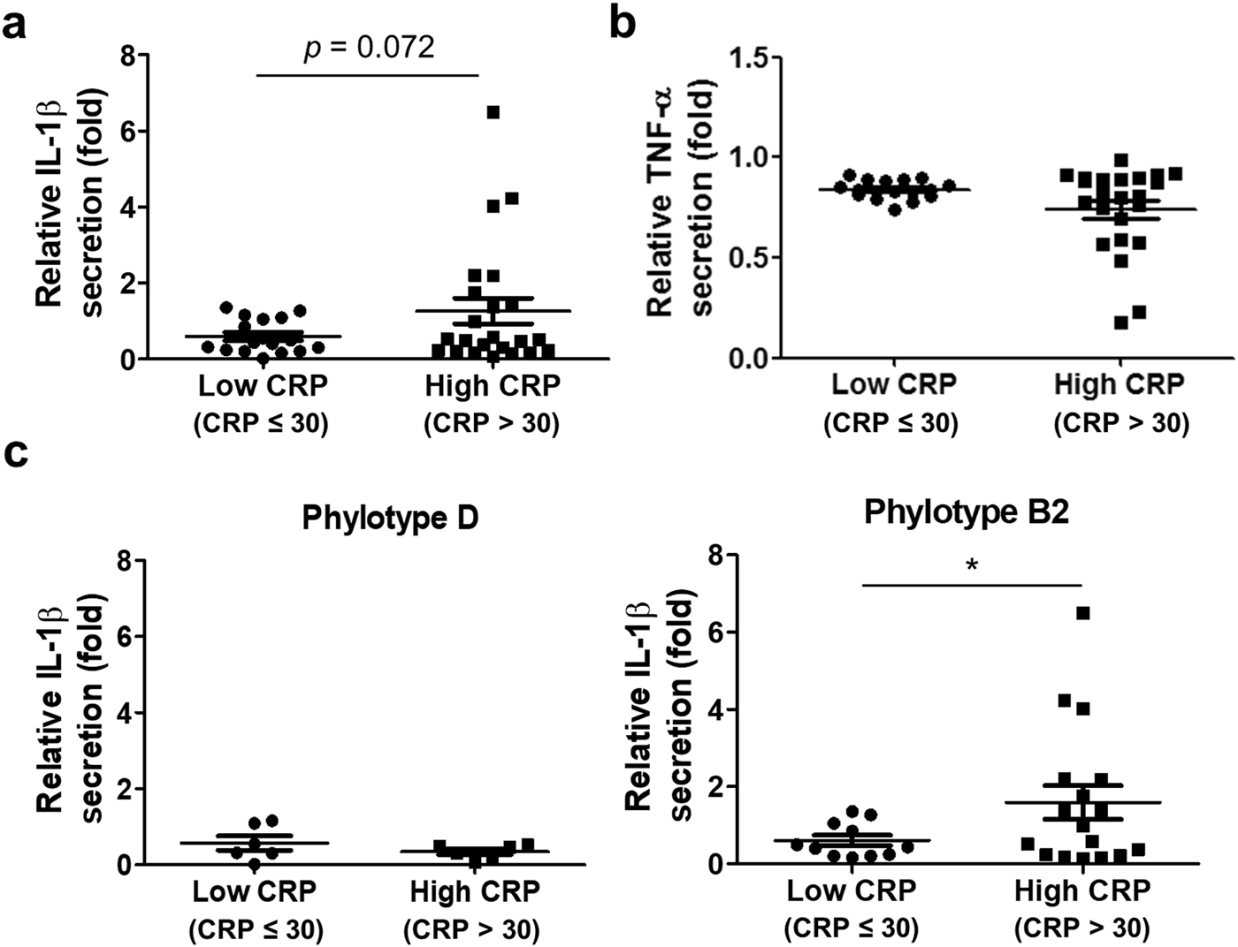
IL-1β induction by group B2 UPEC strains is correlated with patient serum CRP level. (**a**) IL-1β and (**b**) TNF-α induction levels by UPEC isolates were grouped by patient CRP values. (**c**) Two CRP groups were subdivided by phylotype to compare IL-1β induction by UPEC isolates. Each dot represents a mean of six samples pooled from two independent experiments. *p < 0.05.

We then sought to determine which UPEC group might be more associated with IL-1β induction. We also tried to determine the relationship between IL-1β induction potential and patient inflammatory status using the serum CRP level. When the amount of IL-1β secreted by UPEC-treated BMDM was compared between groups B2 and D, group B2 induced significantly more IL-1β secretion (*p* = 0.018) than group D (Fig. 3a). However, we could not detect any significant difference in TNF-α secretion (Fig. 3b). We then divided patients into “high” and “low” inflammation groups (CRP > 30 and <30 mg/L, respectively). UPEC isolates from the high inflammation group induced more IL-1β secretion *in vitro* (Fig. 4a; not statistically significant). Likewise, we found no significant correlation between TNF-α secretion and CRP level (Fig. 4b). We then further analyzed IL-1β secretion between UPECs isolated from the high and low CRP groups after subdividing patients by phylotype. The group B2 UPECs from patients in the high CRP group had significantly more IL-1β induction (*p* = 0.045); however, there was no significant difference in group D UPECs from high and low CRP patients (Fig. 4c). These data suggest that some group B2 UPEC, but not group D UPEC, can augment inflammation, probably by enhancing IL-1β induction during infection.

### Selective virulence genes are involved in IL-1β induction by group B2 UPEC

**Figure 5:**
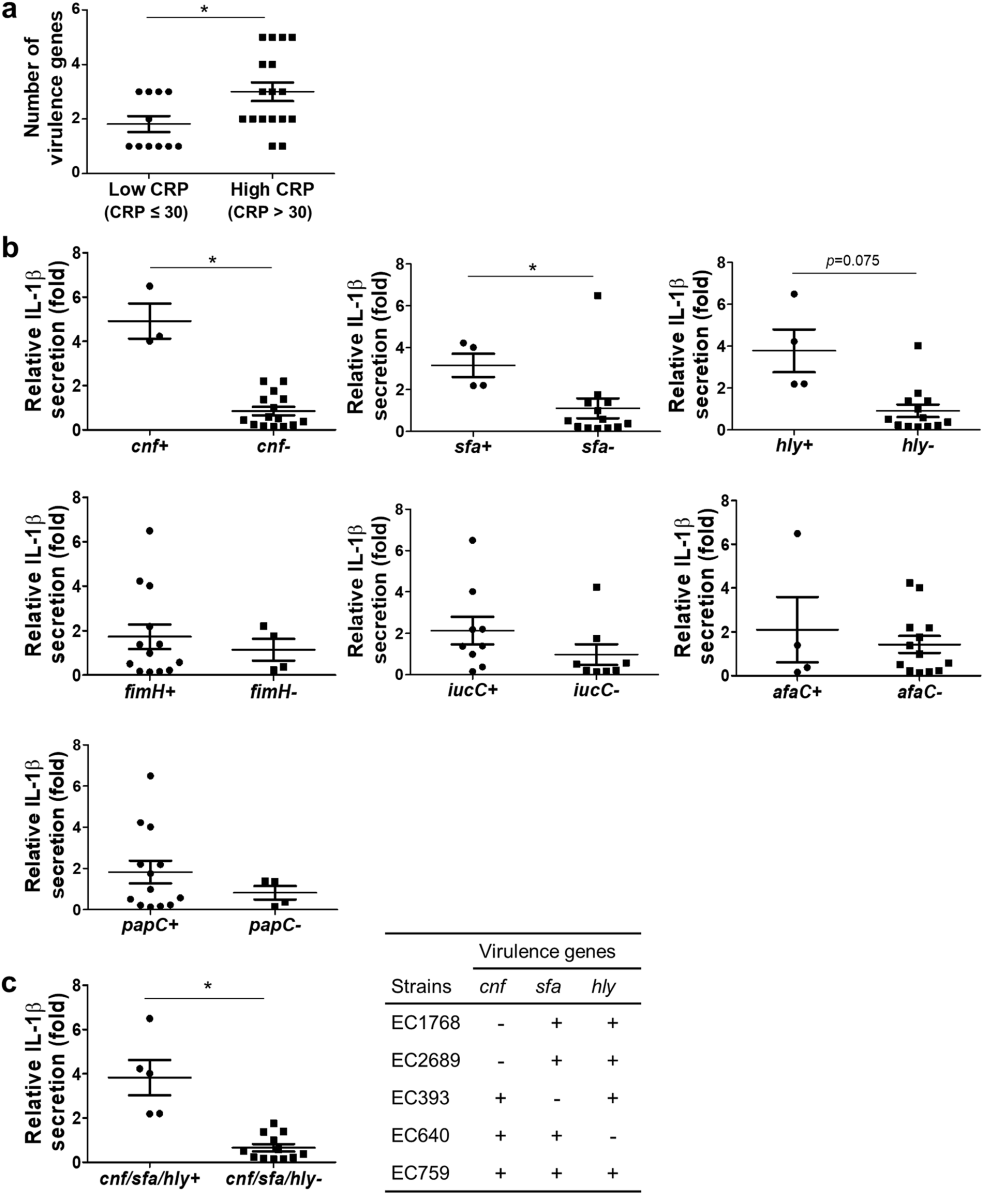
Multiple virulence genes in group B2 UPEC contribute to IL-1β induction in BMDM. (**a**) Virulence gene numbers of UPEC isolates were grouped based on serum CRP level. (**b**) Seven virulence genes were examined in UPECs isolated from patients with serum CRP concentration >30 mg/L (high CRP). (**c**) Strains that possessed *hlyCA, cnf*, and *sfa/focDE* were grouped and compared with other isolates from the high CRP group (CRP > 30 mg/L) for IL-1β induction. Each dot represents a mean of six samples from two independent experiments. *p < 0.05.

It is clear that group B2 UPECs have greater ability to induce IL-1β in macrophages than group D UPECs. However, the amount of IL-1β secretion varied within group B2 (Fig. 4c). We hypothesized that genetic differences in this group might be linked to the observed variation and performed experiments to detect virulence genes from group B2 UPEC isolates. We tested the presence of seven virulence genes by PCR and counted the number of positive genes for each isolate. When group B2 UPECs were divided into high (CRP > 30 mg/L) and low inflammation groups, more virulence genes were detected (*p* = 0.015) in the patients with high CRP levels (Fig. 5a). To further define the virulence gene directly involved in IL-1β induction, we divided UPEC isolates from patients with CRP >30 mg/L based on the presence of each virulence gene and compared *in vitro* IL-1β secretion. Statistical analysis revealed that five isolates (EC1768, EC2689, EC393, EC640, EC759) that possessed at least one *cnf* (*p* = 0.038), *hlyCA* (*p* = 0.075), or *sfa/focDE* (*p* = 0.026) gene induced higher IL-1β secretion than the other isolates (Fig. 5b). However, the presence of the *afaC, fimH, iucC*, or *papC* genes did not significantly affect the amount of IL-1β secreted by macrophages (Fig. 5b). Of note, all five isolates were dual or triple positive for *cnf*, *hlyCA*, and *sfa/focDE* genes. In addition, they induced significantly higher levels of IL-1β secretion (*p* = 0.017) than the other isolates (Fig. 5c). Overall, our data suggest that patients infected with group B2 UPECs that express hemolysin (*hly*), cytotoxic necrotizing factor (*cnf*), S fimbrial adhesion (*sfa*), and F1C fimbriae (*foc*) have stronger inflammatory potential.

## Discussion

Previous research mainly investigated the correlation between cytokine profile and clinical findings, including CRP to assess the contribution of inflammatory components (i.e., inflammatory cytokines) during UTI pathogenesis^36–38^. Although UPECs are the most frequent causative agents of UTI and therefore closely related to disease outcome, most studies did not examine the genetic characteristics of UPECs to link their viral properties with the host’s inflammatory responses. We believe our study is the first to identify genes associated with IL-1β induction in UPECs isolated from young children with UTIs (age < 24 months) and to phylotypically characterize genetic correlation with each child’s serum CRP level. *E. coli* strains belonging to groups B2 and D are considered to be more pathogenic^34,39^. Group B2 UPECs are known to more prevalent than group D UPECs in different age groups and harbor virulence genes including *hlyCA, cnf*, and *sfa/focDE*^39–42^. These virulence genes were exclusively found in group B2 isolates in our study. Our data suggest that phylotypic distribution and pathotypic characteristics of UPEC isolated from Korean patients are similar to those of previous reports^39–42^.

We also pathotyped DH5α, a non-pathogenic group A *E. coil* strain that is commonly used in the laboratory (data not shown). Although DH5α contained *fimH*, it failed to induce IL-1β production by BMDM. However, similar levels of TNF-α were detected upon stimulation, comparable to that of TNF-α induced by group B2 UPEC isolates (data not shown). Consistent with a previous report^17^, IL-1β induction in macrophages was more selective and virulence factor-dependent compared to TNF-α induction. IL-1β, but not TNF-α, showed significant correlation with patient CRP levels. Thus, increased inflammation by group B2 UPEC might be partially attributed to virulence genes related to IL-1β.

Production of IL-1β via caspase-1 signaling can induce a unique type of cell death called ‘pyroptosis’^43^. Pyroptosis can stimulate exfoliation of epithelial cells in urinary tract tissue and possibly give UPECs a chance to reach the inner part of the urothelial layer^44^. During *in vitro* stimulation of BMDM by a UPEC isolate, IL-1β production induces pyroptosis, which may lead to reduced cell survival. Indeed, BMDM treated with group B2 UPEC isolates that expressed *hlyCA, cnf*, and *sfa/focDE* genes showed markedly reduced cell survival. Generally, severe UTI results in overall elevation of inflammatory cytokines^12,28^. However, we observed reduced TNF-α production by BMDM in response to UPECs that induced higher levels of IL-1β. This was probably due to rapid cell death by pyroptosis of BMDM, limiting TNF-α accumulation in culture medium.

Results of our study suggest that UPECs harboring multiple IL-1β-inducible genes are more inflammatory and pathogenic. Three specific genes (i.e., *hlyCA, cnf, sfa/focDE*) were found in the five most IL-1β-inducible strains. Many previous studies have shown the important role of hemolysin and CNF in IL-1β secretion by macrophages upon infection *via* pathways that include activation of Rho GTPase, NF-κB, inflammasomes, and pyroptosis^20,45–48^. Hemolysin encoded by various organisms is well known to activate NLRP3 inflammasome to produce the active form of IL-1β^49^. However, we could not rule out other virulence genes associated with IL-1β induction because minor differences were observed in IL-1β levels from the other 35 UPEC isolates.

We also found that S and F1C fimbriae were associated with IL-1β induction by macrophages. S and F1C fimbriae are known to recognize and bind to sialic acid moieties or gangliotriaosylceramide present on the cell surface of macrophages^50–52^. Bacterial interaction with the urinary tract is an important step to colonization in the bladder. Such interaction is mediated by different types of fimbriae^53^. Some studies have highlighted a pro-inflammatory role of fimbriae^27,54^. Further study is needed to determine the role of S and F1C fimbriae in inflammatory modulation.

Collection bag specimen urine is associated with higher contamination than clean-catch urine or catheter specimen urine^55^. The specimens used in this study were collected between 2010 and 2014 before our laboratory discontinued this practice in 2016^56^. Therefore, some samples were collected with sterile and sealed urine collection bags from febrile infants. Because collection bags have increased contamination risk, the samples used in this study all had a single uropathogenic organism isolated with a colony-forming unit (cfu) count >100,000. We believe this selection criteria helped us to exclude contaminated specimens.

Overall, our results indicate that group B2 UPECs have a greater potential to induce IL-1β. Our data also suggest that IL-1β-inducible genes may play a significant role in the pathogenesis of UTI. However, some patients with group B2 or group D UPECs without noticeable induction of IL-1β *in vitro* also had CRP levels of >30 mg/L. Moreover, many *E. coli* in urinary organs are avirulent^57^. Thus, we suggest that virulence genes need to be examined in addition to detection of bacteria. As multiple virulence factors may synergistically affect disease prognosis, we cannot easily define which virulence factors are important for the pathogenesis of UTI^58,59^. Further studies are required to understand the relationship between underlying mechanisms and the interactions of multiple viral pathways.

## Methods

### Mice

Six- to ten-week-old female WT C57BL/6 (B6) mice were bred and kept under specific pathogen-free conditions in the animal facility of Wide River Institute of Immunology, Seoul National University College of Medicine (Hongcheon, Korea). Animal studies were conducted under protocols approved by the Seoul National University Institutional Animal Care and Use Committee (approval No. SNU-180108-2). All experiments were performed in accordance with relevant guidelines and regulations.

### Subjects and bacterial strains

*P. mirabilis* was kindly provided by Dr. Harry Mobley, University of Michigan, Ann Arbor, USA. *E. coli* strains were isolated from patient urine samples and susceptibility to antimicrobials was tested by Vitek-2 system (BioMérieux, Durham, NC, USA). The clinical samples were then collected and banked at Gyeongsang National University Hospital Branch of the National Culture Collection for Pathogens (GNUH-NCCP, Jinju, Korea). The GNUH Institutional Review Board approved this study (2018-09-012). We retrospectively analyzed data for the 40 pediatric patients under age 24 months with febrile UTI. All had been admitted to Gyeongsang National University Hospital between January 2010 and December 2014. The criteria for diagnosis of a first-time febrile UTI for inclusion in this study have been described^60^. In brief, each child had the following findings: (1) temperature ≥ 38 °C, (2) pyuria (≥5 WBC/high-power field), (3) bacteria-positive urine culture, and (4) no previous history of UTI, kidney, or bladder disease. Renal USG, VCUG, or DMSA scan were used for patient evaluation. Clinical data including age, gender, and WBC counts were recorded. Peripheral venous blood was collected to measure CRP levels by the latex-enhanced turbidimetric assay method (cobas 8000 analyzer; Roche, Indianapolis, IN, USA). Urinary examinations were performed at hospital admission before antibiotic administration or fluid therapies. UTI, USG, and DMSA scan data were collected within 5 days of hospitalization, and VCUG was performed within 4 weeks following antibiotics therapy. The 40 non-duplicate *E. coli* isolates studied were obtained from the GNUH-NCCP and the *E. coli* was identified using PCR with primers specific for *E. coli* alkaline phosphatase gene^61^. Bacteria count was determined by CFU assay on Luria-Bertani (LB) agar plates; optical density (OD) was measured by Epoch spectrophotometer (Bio-Tek, Winooski, VT, USA).

### Bacterial DNA extraction

*E. coli* strains were grown in Müller’s LB broth (BD Difco, Franklin Lakes, NJ, USA) at 37 °C for 18 h. DNA extraction was performed by optimized heat shock method. Bacteria were pelleted from 200 μl of broth, suspended in 200 μl of sterile distilled water, and incubated at 95 °C for 5 min followed by 10 min on ice and centrifugation. We stored 150 μl of the supernatant at −20 °C as a template DNA stock.

### Identification of phylotype and virulence gene distribution by PCR

Specific primers were used to amplify *phoA*^61^*, chuA, yjaC*, and TSPE4.C2 genes^34^ and *fimH, papC, sfa/focDE, afaC, hlyCA, cnf*, and *iucC* operons^62^. Additional information on PCR primers and conditions is summarized in Table 3. For phylotyping, three genes (*chuA, yjaC*, TSPE4.C2) were amplified by multiplex PCR. Phylotype group was categorized according to the combination of these three genes: phylogenetic group A (−/−/+; −/+/−), group B1 (−/−/+), group B2(+/+/−; +/+/+), and group D (+/−/−; +/−/+)^34^. All PCR reactions were carried out by using a 20-μl mixture containing 2 μl of DNA, 10 μl of Topsimple nTaq-Hot premix (Enzynomics, Daejeon, Korea), and 50 pmol of the selected primers in a Veriti thermal cycler (Applied Biosystems, Foster City, CA, USA). The PCR conditions for phylotyping, *phoA* identification, and pathotyping were as follows: (1) for phylotyping, denaturation for 10 min at 94 °C; 35 cycles of 30 s at 94 °C, 30 s at 55 °C, and 30 s at 72 °C; and a final extension step of 10 min at 72 °C; (2) for *phoA* identification, denaturation for 10 min at 94 °C; 35 cycles of 60 s at 94 °C, 60 s at 56 °C, and 60 s at 72 °C; and a final extension step of 10 min at 72 °C; (3) for pathotyping, denaturation for 10 min at 94 °C; 35 cycles of 120 s at 94 °C, annealing s shown in Table 3, and 60 s at 72 °C; and a final extension step of 10 min at 72 °C. Then 10 μl of PCR product was mixed with 1 μl of Midori Green Direct (Nippon Genetics Europe, Dueren, Germany) and followed by 2% agarose gel electrophoresis. Imaging was performed using a Gel-doc XR+ gel documentation system (Bio-Rad, Hercules, CA, USA). Sizes of amplicons were assessed by comparing them with a 1 kb plus DNA ladder (Enzynomics, Daejeon, Korea) on the same gel.

**Table 3:** List of primers and PCR conditions used in this study.

Purpose	Target	Primer	Sequence (5’ to 3’)	Annealing (°C/sec)	Denaturation (sec)	Extension (sec)	Size (bp)	Reference
*E. coli* identification	*E. coli* alkaline phosphatase (*PhoA*)	PhoA-F	GTCACAAAAGCCCGGACACCATAAATGCCT	56/60	60	60	903	^61^
		PhoA-R	TACACTGTCATTACGTTGCGGATTTGGCGT					
Phylotyping	Outer membrane hemin receptor (*chuA*)	ChuA-F	GACGAACCAACGGTCAGGAT	55/30	30	30	279	^34^
		ChuA-R	TGCCGCCAGTACCAAAGACA					
	Conserved stress-induced protein (*yjaA*)	YjaA-F	TGAAGTGTCAGGAGACGCTG	55/30	30	30	211	^34^
		YjaA-R	ATGGAGAATGCGTTCCTCAAC					
	TspE4.C2 fragment	TspE4.C2-F	GAGTAATGTCGGGGCATTCA	55/30	30	30	152	^34^
		TspE4.C2-R	CGCGCCAACAAAGTATTACG					
UPEC pathotyping	Type 1 fimbriae (*fimH*)	FimH-F	AACAGCGATGATTTCCAGTTTGTGTG	65/30	120	60	465	^63^
		FimH-R	ATTGCGTACCAGCATTAGCAATGTCC					
	P fimbriae (*papC*)	PapC-F	GACGGCTGTACTGCAGGGTGTGGCG	65/30	120	60	328	^64^
		PapC-R	ATATCCTTTCTGCAGGGATGCAATA					
	S and FIC fimbriae (*sfa/focDE*)	Sfa-F	CTCCGGAGAACTGGGTGCATCTTAC	65/30	120	60	410	^64^
		Sfa-R	CGGAGGAGTAATTACAAACCTGGCA					
	Afa adhesins (*afaC*)	Afa-F	CGGCTTTTCTGCTGAACTGGCAGGC	65/30	120	60	672	^64^
		Afa-R	CCGTCAGCCCCCACGGCAGACC					
	Hemolysin (*hlyCA*)	Hly-F	AGATTCTTGGGCATGTATCCT	65/30	120	60	556	^65^
		Hly-R	TTGCTTTGCAGACTGTAGTGT					
	Cytotoxic necrotizing factor (*cnf*)	Cnf-F	TTATATAGTCGTCAAGATGGA	58/30	120	60	693	^66^
		Cnf-R	CACTAAGCTTTACAATATTGA					
	Aerobactin (*iucC*)	Aer-F	AAACCTGGCTTACGCAACTGT	60/30	120	60	269	^65^
		Aer-R	ACCCGTCTGCAAATCATGGAT					

### *In vitro* BMDM stimulation

Bone marrow cells were isolated from femurs and tibias of 6- to 10-week-old female B6 mice and cultured for 7 days with macrophage differentiation medium containing RPMI 1640 (Hyclone, South Logan, UT, USA) supplemented with 80 ng/ml M-CSF (BioLegend, San Diego, CA, USA), glutamine, sodium pyruvate, 10% heat-inactivated FBS (Hyclone), 1% 100 × penicillin-streptomycin and 15 mM HEPES (Gibco BRL, Gaithersburg, MD, USA). Differentiated macrophages were detached by cell lifter and 2.0 × 10^5^ of BMDMs were seeded into a 48-well plate followed by incubation overnight for attachment. Cells were then treated with *P. mirabilis* or *E. coli* isolates in RPMI 1640 without antibiotics at an MOI of 1 for 3 h followed by the addition of 100 µg/ml gentamicin (Gibco BRL) and additional culture for 9 h. Culture supernatants of infected cells were harvested and stored in −80 °C until use in an ELISA.

### ELISA

Levels of IL-1β and TNF-α in culture supernatants were measured using Duoset mouse ELISA kits (R&D Systems, Minneapolis, MN, USA) according to the manufacturer’s directions. The amounts of cytokine produced by *E. coli* stimulation were normalized to the same cytokine produced by *P. mirabilis* (positive control) and shown as relative cytokine induction.

### Cell viability assay

BMDMs were washed twice with DPBS supplemented with penicillin-streptomycin and 100 µg/ml gentamicin (Gibco BRL) and then treated with EZ-Cytox Enhanced Cell Viability Assay reagent (Daeil Lab Service, Seoul, Korea) at 37 °C for 30 min. Cell culture supernatants were then placed on 96-well plates and absorbance was measured at a wavelength of 450 nm by Epoch microplate spectrophotometer (Bio-Tek).

### Statistical analysis

Data were analyzed using prism 5 software (GraphPad Software, La Jolla, CA, USA). Student’s *t*-test was performed to determine significance for most data, including experiments investigating any relationship between IL-1β and patient clinical data. We used the Chi-square test to investigate relationships between CRP value in patient serum and patient VUR, DMSA, and sonography data. Significance was defined as a *p* value < 0.05.

## Supplementary information

Supplementary Data 1

## Data Availability

All data generated or analyzed during this study are included in this published article (and its Supplementary Information Files).
