# Supplementary material for: Differential interleukin-1β induction by uropathogenic Escherichia coli correlates with its phylotype and serum C-reactive protein levels in Korean infants
Source: Sci Rep. 2019 Oct 30;9:15654. doi: 10.1038/s41598-019-52070-3 (PMC6821743; doi:10.1038/s41598-019-52070-3)
Supplement: Supplementary file 1 — Supplementary Data 1 [file 41598_2019_52070_MOESM1_ESM.pdf]

# Differential interleukin-1β induction by uropathogenic Escherichia coli correlates with its phylotype and serum C-reactive protein levels in Korean infants.

**Jong-Hyeok Jung[1,2†], Hyun Jung Hong[3,4†], Aziz Gharderpour[1,2], Jae Young Cho[3,4], Bum-Seo Baek[1,2], Yong Hur[5], Byoung Choul Kim[6], Donghyun Kim[1,7], Seung-Yong Seong[1,2,5,7], Jae-Young Lim[3,4*] and Sang-Uk Seo[1,2,8*]**

[1]Department of Biomedical Science, Seoul National University College of Medicine, Seoul, Republic of Korea.

[2]Wide River Institute of Immunology, Seoul National University College of Medicine, Hongcheon, Gangwon-do, Republic of Korea.

[3]Department of Pediatrics, Gyeongsang National University School of Medicine, Jinju, Gyeongsangnam-do, Republic of Korea.

[4]Gyeongsang Institute of Health Science, Jinju, Gyeongsangnam-do, Republic of Korea.

[5] Department of Medicine, Seoul National University College of Medicine, Seoul, Republic of Korea

[6]Division of Nano-bioengineering, Incheon National University, Incheon, Republic of Korea.

[7]Department of Microbiology and Immunology, Institute of Infectious Diseases, Seoul National University College of Medicine, Seoul, Republic of Korea.

[8]Mucosal Immunology Laboratory, University of Ulsan College of Medicine, Seoul, Republic of Korea.

*Corresponding authors: Dr. Sang-Uk Seo (suseo@ulsan.ac.kr), Dr. Jae-Young Lim (pedneu@gnu.ac.kr)

**Supplementary Table 1**

| Strains | phoA | phylotype | Pathotyping: papC | sfa/foc | afaC | fimH | iucC | cnf | hly | No. of virulence gene | CRP (mg/L) | WBC/mm$^3$ | DMSA | VUR | Sonograpy | Disease Diagnosis |
|---|---|---|---|---|---|---|---|---|---|---|---|---|---|---|---|---|
| EC1082 | P | D | N | N | N | P | P | N | N | 2 | 38.8 | 24030 | No cortical defect in both kidneys. | - | No remarkable findings. | Acute pyelonephritis |
| EC1280 | P | B2 | P | N | N | N | N | N | N | 1 | 133.1 | 21490 | No cortical defect in right kidney. Multiple cortical defects on left kidney. - REC) F/U scan. | - | R/O Urachal remnant at bladder dome. | Acute pyelonephritis |
| EC129 | P | D | P | N | N | P | P | N | N | 3 | 5.8 | 6680 | No cortical defect in both kidneys. | - | No remarkable findings. | Urinary tract infection |
| EC134 | P | B2 | P | N | N | P | N | N | N | 2 | 36.4 | 10800 | No cortical defect in both kidneys. | - | No remarkable findings. | Acute pyelonephritis |
| EC153 | P | D | N | N | N | P | P | N | N | 2 | 1.5 | 11210 | No cortical defect in both kidneys. | - | No remarkable findings. | Acute pyelonephritis |
| EC168 | P | B2 | P | N | N | P | P | N | N | 3 | 12.1 | 23650 | No cortical defect in both kidneys. | - | Bladder wall thickening, R/O Cystitis | Acute pyelonephritis |
| EC172 | P | B2 | N | N | P | P | P | N | N | 3 | 60.8 | 25090 | No cortical defect in right kidney. Suspicious cortical defect on left kidney upper portion. - REC) F/U. | - | Bladder wall thickening, R/O Cystitis. | Acute pyelonephritis |
| EC1759 | P | B2 | N | N | P | P | P | N | N | 3 | 14.1 | 12990 | Unremarkable renal scan | - | Bladder wall thickening, R/O Cystitis. | Urinary tract infection |
| EC1768 | P | B2 | P | P | N | N | P | N | P | 4 | 42.7 | 15590 | Unremarkable renal scan | - | No remarkable findings. | Urinary tract infection |
| EC211 | P | D | N | N | N | P | P | N | N | 2 | 0.9 | 18810 | No cortical defect in both kidneys. | - | Gall bladder stones. | Acute pyelonephritis |
| EC220 | P | D | N | N | N | P | P | N | N | 2 | 29.4 | 15240 | No cortical defect in both kidneys. | - | No remarkable findings. | Acute pyelonephritis |
| EC2689 | P | B2 | P | P | N | P | P | N | P | 5 | 42.9 | 10240 | Hydronephrosis/reflux of Left kidney | - | No remarkable findings. | Urinary tract infection |
| EC291 | P | D | P | N | N | P | N | N | N | 2 | 41.3 | 10850 | Unremarkable renal scan | Bilateral VUR, grade 1/1 | Mild left hydronephrosis. | Urinary tract infection |
| EC314 | P | B2 | N | N | P | N | P | N | N | 4 | 51.7 | 10460 | Unremarkable renal scan | - | Bladder wall thickening, R/O Cystitis. | Urinary tract infection |
| EC378 | P | B2 | P | N | N | P | N | N | N | 2 | 36.4 | 14790 | No cortical defect in both kidneys. | - | Bladder wall thickening, R/O Cystitis. | Acute pyelonephritis |
| EC388 | P | B2 | P | N | N | P | N | N | N | 2 | 36.7 | 16770 | No cortical defect in right kidney. Possible multiple cortical defects on left kidney. - REC) F/U. | VUR, right. grade 3. | Prominent left renal pelvis. | Acute pyelonephritis |
| EC393 | P | B2 | P | N | P | P | P | P | P | 5 | 37.7 | 13830 | No cortical defect in both kidneys. | - | No remarkable findings. | Acute pyelonephritis |
| EC395 | P | D | P | N | N | P | P | N | N | 3 | 8 | 8920 | No cortical defect in both kidneys. | - | 1. Heterogeneous echogenicity in both kidneys; R/O APN.<br>2. Mild wall thickening in the blader; R/O Cystitis. | Urinary tract infection |
| EC428 | P | D | P | N | N | P | P | N | N | 3 | 60.1 | 19570 | No cortical defect in right kidney. Multiple cortical defects on left kidney. - REC) F/U. | - | No remarkable findings | Acute pyelonephritis |
| EC4500 | P | B2 | P | N | N | N | N | N | N | 1 | 19.8 | 10910 | No cortical defect in both kidneys. | - | No remarkable findings. | Acute pyelonephritis |
| EC53 | P | B2 | P | N | N | N | N | N | N | 1 | 19.4 | 17230 | No cortical defect in both kidneys. | - | Transient small bowel intussusception at LLQ. | Acute pyelonephritis |
| EC563 | P | D | P | N | N | N | P | N | N | 2 | 116.1 | 10810 | No cortical defect in both kidneys. | - | No remarkable findings. | Acute pyelonephritis |
| EC59 | P | B2 | N | N | N | P | N | N | N | 1 | 26.2 | 14800 | No cortical defect in both kidneys. | - | No remarkable findings. | Acute pyelonephritis |
| EC604 | P | B2 | P | N | N | P | N | N | N | 2 | 54.1 | 16530 | No cortical defect in right kidney. Cortical defects on left kidney. - REC) F/U. | VUR, left, grade 1. | Slightly prominent both renal pelvis. | Acute pyelonephritis |
| EC640 | P | B2 | P | P | N | P | P | P | N | 5 | 99.7 | 15620 | No cortical defect in both kidneys. | - | Bladder wall thickening, R/O Cystitis. | Urinary tract infection |
| EC663 | P | B2 | P | N | N | P | P | N | N | 3 | 7.1 | 12240 | No cortical defect in both kidneys. | - | Swelling of Left kidney. Conclusion: R/O APN, left kidney. | Urinary tract infection |
| EC667 | P | B2 | N | N | P | N | N | N | N | 1 | 13 | 9860 | No cortical defect in both kidneys. | - | Normal kidney and bladder. | Acute pyelonephritis |
| EC668 | P | D | P | N | N | P | P | N | N | 3 | 4.2 | 24580 | Unremarkable renal scan | - | Slightly prominent left renal pelvis. | Urinary tract infection |
| EC68 | P | B2 | P | N | N | N | N | N | N | 1 | 100.7 | 22360 | Suspicious cortical defects on both kidneys. - REC) F/U DMSA scan. | - | No remarkable findings. | Acute pyelonephritis |
| EC714 | P | B2 | P | N | N | P | N | N | N | 2 | 32.8 | 14090 | Destructive lesion, probably inflammatory in nature, involvingupper polar area of Lt kidney | - | No remarkable findings. | Acute pyelonephritis |
| EC726 | P | B2 | N | P | N | P | N | N | N | 2 | 2.7 | 6020 | Hydronephrosis mainly affecting Rt kidney, possibly withdiffuse interstitial disease | VUR, both, grade 3. | No remarkable findings. | Urinary tract infection |
| EC731 | P | D | N | N | P | P | N | N | N | 2 | 189.5 | 16710 | Destructive lesions involving upper polar areas of kidneys,<br>more severely in Right kidney which also shows calyectatic feature | | No remarkable findings. | Urinary tract infection |
| EC734 | P | B2 | N | N | N | P | P | N | N | 2 | 52.3 | 15740 | No cortical defect in both kidneys. | - | No remarkable findings. | Urinary tract infection |
| EC751 | P | B2 | P | N | N | P | P | N | N | 3 | 57.3 | 24020 | No cortical defect in right kidney. R/O dilated excretional system such as calyx on left kidney. | - | No remarkable findings. | Acute pyelonephritis |
| EC759 | P | B2 | P | P | N | P | N | P | P | 5 | 51.6 | 13790 | No cortical defect in both kidneys. | - | No remarkable findings | Acute pyelonephritis |
| EC765 | P | B2 | N | N | N | P | N | N | N | 1 | 9.5 | 20790 | Unremarkable renal scan | - | 1. No change of mild left hydronephrosis. 2. Bladder wall thickening, R/O Cystitis. | Urinary tract infection |
| EC770 | P | D | P | N | N | P | P | N | N | 3 | 142.6 | 19010 | No cortical defect in both kidneys. | - | Bladder wall thickening, R/O Cystitis | Urinary tract infection |
| EC868 | P | B2 | N | N | P | P | P | N | N | 3 | 76.5 | 7610 | Hydronephrosis of Left kidney with cicatrical change | - | CT: 1. Wedge shape low density lesion in Left kidney; c/w Left APN.<br>2. Multiple clustered nodular lesion at left lower pararenal area; R/O Benign lesion, such as hemangioma.<br>3. Lower inguinal location of right testis. | Urinary tract infection |
| EC965 | P | B2 | N | P | N | N | N | N | N | 1 | 3.9 | 21490 | The kidneys may be affected by diffuse interstitial disease. | - | 1. Nonspecific bright echo Vs tiny renal stone at left kidney.<br>2. Otherwise no remarkable findings. | Acute pyelonephritis |
| EC981 | P | B2 | P | N | N | P | P | N | N | 3 | 12.9 | 4470 | Unremarkable renal scan | 1. VUR, left, grade 1.<br>2. Normal shaped bladder and urethra | Normal kidney and bladder. | Urinary tract infection |

| Strains | Cell viability (Average) | IL-1β (pg/ml): Experiment 1 (Average) | IL-1β: Experiment 2 (Average) | IL-1β: Average | IL-1β: SD | Relative IL-1β (Fold of *P. mirabilis* group): Experiment 1 (Average) | Relative IL-1β: Experiment 2 (Average) | Relative IL-1β: Average | Relative IL-1β: SD | TNF-α (pg/ml): Experiment 1 (Average) | TNF-α: Experiment 2 (Average) | TNF-α: Average | TNF-α: SD | Relative TNF-α (Fold of *P. mirabilis* group): Experiment 1 (Average) | Relative TNF-α: Experiment 2 (Average) | Relative TNF-α: Average | Relative TNF-α: SD |
|---|---|---|---|---|---|---|---|---|---|---|---|---|---|---|---|---|---|
| EC1082 | 111.9517 | 329.298 | 119.8291 | 224.5636 | 148.1169 | 0.837887077 | 0.206289155 | 0.522 | 0.446607 | 6102.919 | 7454.452 | 6778.686 | 955.6781 | 0.919043095 | 0.918750821 | 0.918897 | 0.000207 |
| EC1280 | 113.6 | 1069.520733 | 903.2623333 | 986.3915 | 117.5624 | 1.613638428 | 1.901240675 | 1.757 | 0.203365 | 6934.4264 | 7382.066533 | 7158.246 | 316.5294 | 0.959680614 | 1.012998762 | 0.98634 | 0.037702 |
| EC129 | 115.8545 | 139.4876667 | 423.8743333 | 281.681 | 201.0917 | 0.210451889 | 0.892196092 | 0.551 | 0.482066 | 5654.922667 | 6362.41 | 6008.666 | 500.2691 | 0.782605416 | 0.873077129 | 0.827841 | 0.063973 |
| EC134 | 114.876 | 385.046 | 103.1395 | 244.0928 | 199.338 | 0.979735885 | 0.177557541 | 0.579 | 0.567226 | 5693.0115 | 7484.878 | 6588.945 | 1267.041 | 0.857314821 | 0.922500783 | 0.889908 | 0.046093 |
| EC153 | 100.1455 | 766.5143333 | 485.3343333 | 625.9243 | 198.8243 | 1.156477799 | 1.021560782 | 1.089 | 0.095401 | 5410.436933 | 7079.404133 | 6244.921 | 1180.138 | 0.748770142 | 0.971466132 | 0.860118 | 0.15747 |
| EC168 | 97.96567 | 969.4045 | 41.5966 | 505.5006 | 656.0593 | 2.466615353 | 0.071609713 | 1.269 | 1.693525 | 5328.8105 | 6952.884 | 6140.847 | 1148.393 | 0.802469523 | 0.856933264 | 0.829701 | 0.038512 |
| EC172 | 100.9455 | 471.6423333 | 980.0873333 | 725.8648 | 359.5249 | 0.711589939 | 2.062946537 | 1.387 | 0.955553 | 1439.8864 | 6826.030267 | 4132.958 | 3808.579 | 0.199271142 | 0.936697086 | 0.567984 | 0.521439 |
| EC1759 | 106.2396 | 2388.768933 | 337.438 | 1363.103 | 1450.51 | 0.201 | 0.796 | 0.498 | 0.421018 | 8637.9436 | 7687.745767 | 8162.845 | 671.8913 | 0.925 | 0.906 | 0.915262 | 0.013117 |
| EC1768 | 2.412646 | 6214.329617 | 3940.0588 | 5077.194 | 1608.152 | 2.342 | 2.071 | 2.206 | 0.191739 | 8112.7959 | 5354.9495 | 6733.873 | 1950.092 | 0.644 | 0.851 | 0.747449 | 0.14631 |
| EC211 | 98.09282 | 591.61225 | 467.5291 | 529.5707 | 87.74004 | 1.505336378 | 0.804864453 | 1.155 | 0.495308 | 6006.4275 | 7280.194 | 6643.311 | 900.6889 | 0.904512369 | 0.897273765 | 0.900893 | 0.005118 |
| EC220 | 106.1818 | 87.9698 | 225.1536667 | 156.5617 | 97.00364 | 0.132724355 | 0.473916927 | 0.303 | 0.24126 | 5852.555733 | 6230.7172 | 6041.636 | 267.4005 | 0.809956578 | 0.855005679 | 0.832481 | 0.031855 |
| EC2689 | 6.738769 | 4732.822567 | 4711.8592 | 4722.341 | 14.82334 | 2.801 | 1.577 | 2.189 | 0.865186 | 4867.4026 | 5516.436583 | 5191.92 | 458.9363 | 0.663 | 0.511 | 0.586964 | 0.108115 |
| EC291 | 95.34109 | 1053.1174 | 435.7396 | 744.4285 | 436.552 | 0.259 | 0.351 | 0.305 | 0.064999 | 8344.6793 | 7873.225217 | 8108.952 | 333.3684 | 0.947 | 0.875 | 0.911035 | 0.05064 |
| EC314 | 88.17218 | 628.5705 | 699.4991667 | 664.0348 | 50.15414 | 0.598 | 0.155 | 0.377 | 0.313835 | 14925.84533 | 10962.65683 | 12944.25 | 2802.397 | 1.036 | 0.786 | 0.910703 | 0.176671 |
| EC378 | 96.58182 | 41.197 | 133.988 | 87.5925 | 65.61315 | 0.062155936 | 0.28202597 | 0.172 | 0.155472 | 4972.577467 | 6121.358267 | 5546.968 | 812.3107 | 0.68817317 | 0.839998978 | 0.764086 | 0.107357 |
| EC388 | 98.03636 | 0 | 130.915 | 65.4575 | 92.57088 | 0 | 0.275557735 | 0.138 | 0.194849 | 5470.919467 | 6427.871333 | 5949.395 | 676.6672 | 0.757140541 | 0.882060013 | 0.8196 | 0.088331 |
| EC393 | 25.74545 | 4793.042333 | 2737.843333 | 3765.443 | 1453.245 | 7.231498235 | 5.762776675 | 6.497 | 1.038543 | 1765.299467 | 808.2084 | 1286.754 | 676.7656 | 0.244306245 | 0.110905815 | 0.177606 | 0.094328 |
| EC395 | 118.9091 | 50.00926667 | 267.1513333 | 158.5803 | 153.5426 | 0.075451435 | 0.562316132 | 0.319 | 0.344265 | 5548.439333 | 5970.412133 | 5759.426 | 298.3798 | 0.767868798 | 0.819285504 | 0.793577 | 0.036357 |
| EC428 | 95.04132 | 93.3645 | 119.8291 | 106.5968 | 18.7133 | 0.237562657 | 0.206289155 | 0.222 | 0.022114 | 5587.0885 | 7542.042 | 6564.565 | 1382.361 | 0.841363798 | 0.929546167 | 0.885455 | 0.062354 |
| EC4500 | 136.5225 | 509.6760333 | 540.019 | 524.8475 | 21.45572 | 0.321 | 0.170 | 0.245 | 0.106873 | 8121.5646 | 6474.285683 | 7297.925 | 1164.802 | 0.779 | 0.852 | 0.815215 | 0.051775 |
| EC53 | 107.883 | 552.78775 | 171.2887 | 362.0382 | 269.7606 | 1.406548816 | 0.294878299 | 0.851 | 0.78607 | 5637.148 | 7558.638 | 6597.893 | 1358.699 | 0.848902295 | 0.9315916 | 0.890247 | 0.05847 |
| EC563 | 118.9447 | 276.03875 | 127.4785 | 201.7586 | 105.048 | 0.702370805 | 0.219457812 | 0.461 | 0.341471 | 4724.469 | 6785.08 | 5754.775 | 1457.072 | 0.711461288 | 0.836251655 | 0.773856 | 0.08824 |
| EC59 | 107.1837 | 211.33125 | 198.757 | 205.0441 | 8.891337 | 0.537724867 | 0.342165748 | 0.440 | 0.138281 | 5580.559 | 7518.07 | 6549.315 | 1370.027 | 0.840380516 | 0.926591651 | 0.883486 | 0.06096 |
| EC604 | 118.6268 | 372.60225 | 42.9874 | 207.7948 | 233.0729 | 0.948073204 | 0.074004014 | 0.511 | 0.61806 | 5683.58 | 7491.332 | 6587.456 | 1278.274 | 0.855894524 | 0.92329623 | 0.889595 | 0.04766 |
| EC640 | 19.70909 | 3050.925 | 1632.587667 | 2341.756 | 1002.916 | 4.603080302 | 3.436368331 | 4.020 | 0.82499 | 3342.956533 | 3720.082533 | 3531.52 | 266.6684 | 0.462643973 | 0.510485646 | 0.486565 | 0.033829 |
| EC663 | 107.3455 | 0 | 193.9115 | 96.95575 | 137.1161 | 0 | 0.408156543 | 0.204 | 0.28861 | 5792.925067 | 5958.860133 | 5875.893 | 117.3338 | 0.801704072 | 0.817700289 | 0.809702 | 0.011311 |
| EC667 | 111.8881 | 1141.626 | 472.3969 | 807.0115 | 473.2164 | 1.904826849 | 0.813244507 | 1.359 | 0.771865 | 5521.7935 | 7708.002 | 6614.898 | 1545.883 | 0.831530975 | 0.950000505 | 0.890766 | 0.083771 |
| EC668 | 110.2683 | 70.8884 | 0 | 35.4442 | 50.12567 | 0.039 | 0.000 | 0.020 | 0.027577 | 6301.3208 | 6210.519333 | 6255.92 | 64.20633 | 0.795 | 0.679 | 0.736927 | 0.082127 |
| EC68 | 94.98182 | 22.8946 | 200.5696667 | 111.7321 | 125.6352 | 0.034542207 | 0.422171051 | 0.228 | 0.274095 | 6172.005733 | 6839.892667 | 6505.949 | 472.2674 | 0.854166431 | 0.938599344 | 0.896383 | 0.059703 |
| EC714 | 94.63364 | 578.6924 | 134.92385 | 356.8081 | 313.7918 | 0.322 | 0.117 | 0.220 | 0.144799 | 2066.268217 | 1823.506633 | 1944.887 | 171.6584 | 0.261 | 0.199 | 0.230161 | 0.043613 |
| EC726 | 141.2281 | 641.7832 | 55.5507 | 348.667 | 414.529 | 0.358 | 0.048 | 0.203 | 0.219017 | 6687.052767 | 6479.792067 | 6583.422 | 146.5554 | 0.844 | 0.708 | 0.776144 | 0.095963 |
| EC731 | 124.8752 | 237.2149667 | 88.363 | 162.789 | 105.2542 | 0.053 | 0.079 | 0.066 | 0.018755 | 8308.6302 | 7698.819167 | 8003.725 | 431.2015 | 0.926 | 0.871 | 0.898658 | 0.038482 |
| EC734 | 85.09091 | 1376.594333 | 323.4896667 | 850.042 | 744.6575 | 2.076935441 | 0.680900431 | 1.379 | 0.987146 | 2384.606533 | 5971.182267 | 4177.894 | 2536.092 | 0.330014414 | 0.819391185 | 0.574703 | 0.346042 |
| EC751 | 96.56707 | 664.28375 | 167.464 | 415.8739 | 351.3046 | 1.690246431 | 0.288293971 | 0.989 | 0.99133 | 5383.223 | 7615.802 | 6499.513 | 1578.672 | 0.810663541 | 0.938636984 | 0.87465 | 0.090491 |
| EC759 | 20.66116 | 1376.06625 | 2878.1332 | 2127.1 | 1062.122 | 3.501351747 | 4.954786993 | 4.228 | 1.027734 | 5147.4355 | 4985.336 | 5066.386 | 114.6217 | 0.775156127 | 0.614435715 | 0.694796 | 0.113646 |
| EC765 | 121.7234 | 2986.145 | 507.08405 | 1746.615 | 1752.961 | 1.663 | 0.441 | 1.052 | 0.864397 | 7165.2182 | 7091.654367 | 7128.436 | 52.01749 | 0.905 | 0.775 | 0.840085 | 0.091804 |
| EC770 | 133.1214 | 121.73625 | 387.5581 | 254.6472 | 187.9644 | 0.309753569 | 0.667192134 | 0.488 | 0.252747 | 5465.93 | 6410.748 | 5938.339 | 668.0872 | 0.82311845 | 0.790115758 | 0.806617 | 0.023336 |
| EC868 | 130.3922 | 304.786 | 159.10375 | 231.9449 | 103.0129 | 0.170 | 0.138 | 0.154 | 0.022465 | 6824.814183 | 6685.0792 | 6754.947 | 98.80755 | 0.862 | 0.731 | 0.796364 | 0.092824 |
| EC965 | 114.8918 | 589.6374333 | 1033.5196 | 811.5785 | 313.8721 | 0.614 | 0.196 | 0.405 | 0.295444 | 7557.4449 | 7598.235783 | 7577.84 | 28.84351 | 0.914 | 0.793 | 0.853216 | 0.08564 |
| EC981 | 110.9817 | 638.5027333 | 193.9708 | 416.2368 | 314.3315 | 0.115 | 0.213 | 0.164 | 0.06892 | 7678.2581 | 7282.643883 | 7480.451 | 279.7415 | 0.876 | 0.805 | 0.840575 | 0.049843 |

P; Positive, N; Negative
